# Chronic Fluoxetine Induces the Enlargement of Perforant Path-Granule Cell Synapses in the Mouse Dentate Gyrus

**DOI:** 10.1371/journal.pone.0147307

**Published:** 2016-01-20

**Authors:** Yosuke Kitahara, Keisuke Ohta, Hiroshi Hasuo, Takahide Shuto, Mahomi Kuroiwa, Naoki Sotogaku, Akinobu Togo, Kei-ichiro Nakamura, Akinori Nishi

**Affiliations:** 1 Department of Pharmacology, Kurume University School of Medicine, 67 Asahi-machi, Kurume, Fukuoka, 830–0011, Japan; 2 Department of Anatomy, Kurume University School of Medicine, 67 Asahi-machi, Kurume, Fukuoka, 830–0011, Japan; 3 Department of Physiology, Kurume University School of Medicine, 67 Asahi-machi, Kurume, Fukuoka, 830–0011, Japan; Nathan Kline Institute and New York University School of Medicine, UNITED STATES

## Abstract

A selective serotonin reuptake inhibitor is the most commonly prescribed antidepressant for the treatment of major depression. However, the mechanisms underlying the actions of selective serotonin reuptake inhibitors are not fully understood. In the dentate gyrus, chronic fluoxetine treatment induces increased excitability of mature granule cells (GCs) as well as neurogenesis. The major input to the dentate gyrus is the perforant path axons (boutons) from the entorhinal cortex (layer II). Through voltage-sensitive dye imaging, we found that the excitatory neurotransmission of the perforant path synapse onto the GCs in the middle molecular layer of the mouse dentate gyrus (perforant path-GC synapse) is enhanced after chronic fluoxetine treatment (15 mg/kg/day, 14 days). Therefore, we further examined whether chronic fluoxetine treatment affects the morphology of the perforant path-GC synapse, using FIB/SEM (focused ion beam/scanning electron microscopy). A three-dimensional reconstruction of dendritic spines revealed the appearance of extremely large-sized spines after chronic fluoxetine treatment. The large-sized spines had a postsynaptic density with a large volume. However, chronic fluoxetine treatment did not affect spine density. The presynaptic boutons that were in contact with the large-sized spines were large in volume, and the volumes of the mitochondria and synaptic vesicles inside the boutons were correlated with the size of the boutons. Thus, the large-sized perforant path-GC synapse induced by chronic fluoxetine treatment contains synaptic components that correlate with the synapse size and that may be involved in enhanced glutamatergic neurotransmission.

## Introduction

Major depressive disorder is one of the most common psychiatric disorders, but the efficacy of antidepressant medication is not sufficient [1, 2]. The monoamine and monoamine receptor hypotheses of depression were derived from analyses of antidepressant action [3], but the mechanisms underlying its action are not fully understood [2, 4]. The induction of adult neurogenesis in the hippocampal dentate gyrus (DG) has been implicated in the therapeutic action of antidepressants [5–7], and the upregulation of brain-derived neurotrophic factor (BDNF) [8] contributes to the neurogenesis [8–10] as well as to the dendritic outgrowth and synaptic plasticity [11] induced by antidepressants. Antidepressants were recently shown to increase the excitability of mature granule cells (GCs) in the DG and to reduce the expression of mature GC markers such as calbindin and tryptophan-2,3-dioxygenase [12]. These observations suggest that the DG may be one of the therapeutic targets of antidepressants.

The DG receives excitatory inputs from the entorhinal cortex and functions as the main gateway to the hippocampus. In the molecular layer of the DG, the perforant path from layer II of the entorhinal cortex (medial subdivision) forms a synapse onto the dentate GCs (perforant path-GC synapse). Chronic treatment with fluoxetine, one of the selective serotonin reuptake inhibitors (SSRIs), induces an increase in the excitability of the dentate GCs, as previously described [12], and alterations in the synaptic plasticity at perforant path-GC synapses [12–14]. It has been reported that long-term potentiation (LTP) at perforant path-GC synapses is enhanced by chronic fluoxetine under conditions of intact synaptic inhibition [14], although reduced LTP and enhanced long-term depression (LTD) under disinhibited conditions with a GABA_A_ receptor inhibitor are reported [12]. It has been proposed that synaptic responses to glutamatergic stimulation, which are determined as excitatory postsynaptic currents (EPSCs), are positively correlated with the spine volumes [15], although conflicting findings on the correlation of synaptic function with structure have been reported [16, 17]. Furthermore, an alteration of synaptic plasticity is known to affect synaptic morphology and density, with larger or more numerous spines in LTP and smaller or fewer spines in LTD [18]. Therefore, it is highly possible that chronic fluoxetine treatment induces morphological changes in the perforant path-GC synapses.

The information on antidepressant-induced alterations of spine morphology and density is limited. Chronic fluoxetine treatment has been reported to increase spine density in the CA1 and CA3 regions of the hippocampus [19, 20]. McAvoy et al. recently reported that chronic fluoxetine treatment induces changes in spine density and size in the DG and CA1 in laminae-specific input- and age-dependent manners [21]. In the DG, increases in spine density and size were detected in the outer molecular layer of the dorsal DG in middle-aged mice (10 months of age) but not in adult mice (4 months of age). Their study clearly demonstrated that chronic fluoxetine treatment induces morphological changes in the dendritic spines of the dentate GCs, but the fine structures of the spines and presynaptic boutons were not evaluated due to limitations in the fluorescent imaging of the dendritic spines.

Imaging with electron microscopy is required for the analysis of the fine structures of the synapse in the nervous system. Due to the complexity of neural connections, three-dimensional (3D) reconstruction using electron microscope images is desired. Serial section transmission electron microscopy (ssTEM) has been used for 3D reconstruction, but ssTEM is time-consuming and technically demanding [22, 23]. To overcome the disadvantages, focused ion beam/scanning electron microscopy (FIB/SEM) has been developed to acquire serial section SEM images, which have been shown to be sufficient for the reconstruction of 3D images of the synaptic connections and structures [22–25]. Recently, the FIB/SEM technique was successfully used to reconstruct 3D structures of the dendritic spines of adult-generated GCs and their contacts with the boutons of the perforant path in the DG [26].

In this study, we performed voltage-sensitive dye imaging and found that glutamatergic neurotransmission at the perforant path-GC synapses in the middle molecular layer of DG was enhanced after chronic fluoxetine treatment. The functional upregulation of the perforant path-GC synapse is likely associated with the reduced expression of mature GC markers [12]. In the dorsal DG where mature GC markers are reduced after chronic fluoxetine, we quantitatively analyzed the chronic fluoxetine treatment-induced changes in the structures of the perforant path-GC synapses using FIB/SEM imaging and 3D reconstruction.

## Materials and Methods

### Animals

Male C57BL/6N mice were purchased from Japan SLC (Shizuoka, Japan). Mice were housed 2–4 per cage and were maintained on a 12-h light/dark cycle (lights on from 7:00 am to 7:00 pm) with access to food and water *ad libitum*. All of the mice used in this study were handled in accordance with the Guide for the Care and Use of Laboratory Animals as adopted by the U.S. National Institutes of Health, and the specific protocols were approved by the Institutional Animal Care and Use Committee of Kurume University School of Medicine (Protocol Numbers 2012–057, 2013–077, 2014–026, and 2015–031).

### Drug treatment

Male C57BL/6N mice at 10 weeks of age were treated with fluoxetine chronically by the subcutaneous implantation of matrix-driven delivery pellets (Innovative Research of America, Sarasota, FL, USA), which were designed to consistently release fluoxetine at a rate of 15 mg/kg/day for 14 days. In the placebo group, the mice received subcutaneous implantation of pellets containing only matrix for the same period.

### Electrophysiology

The neuronal activities of the DG in the hippocampal slices were recorded by the use of a voltage-sensitive dye and an optical recording technique [27], as reported previously [28]. Mice treated with placebo or fluoxetine pellets were sacrificed by decapitation. The brains were rapidly removed and immersed for 8–10 s in a cooled artificial cerebrospinal fluid (ASCF at 4–6°C) that was pre-bubbled with 95% O_2_/5% CO_2_. The composition of the ACSF was as follows (in mM): NaCl 117, KCl 4.7, CaCl_2_ 2.5, NaHPO_4_ 1.2, and D-glucose 11 (299 ± 4 mOsm, pH 7.4). Horizontal brain slices (400 μm in thickness) were cut from the ventro-to-mid section at -2.2 – -3.0 mm from the bregma by a Vibroslice (Campden Instruments, Loughborough, Leics., UK). The brain slices were allowed to recover for 1 hour in the oxygenated ACSF at room temperature (22–24°C), were then transferred to a recording chamber and were continuously superfused with the oxygenated ACSF at a rate of 2–3 ml/min. The temperature of the brain slices was maintained at 28.8–29.2°C in the recording chamber. The slices were submerged in ACSF containing the voltage-sensitive dye RH-482 (0.1 mg/ml) (Nippon Kankoh-Shikiso Kenkyusho, Okayama, Japan) for 15 min. A stained slice was mounted on an upright microscope (Optiphoto-2, Nikon) equipped with a tungsten-halogen lamp (12 V/100 W), interference filters (700 nm) and a mechanical shutter to control the duration of light exposure. Optical images were acquired using a 128x128 photodiode array with a time resolution of 0.6 ms (HR Deltaron 1700, FUJIFILM, Tokyo, Japan). With a 10X objective and a 0.6X relay lens (total 6X), each photodiode monitored an area of 11.5x11.5 μm^2^, and therefore, the whole array monitored a tissue area of 1.48x1.48 mm^2^. The stained slice was illuminated by a 700 nm light for 2 s every 10 s to minimize bleaching of the voltage-sensitive dye. To evoke optical signals, a concentric bipolar electrode was placed in the middle molecular layer of the hippocampal DG, where the perforant path inputs to the GCs from the medial entorhinal cortex were present. Before applying the stimulus, 128 picture frames of the averaged background image were stored as reference light signals. These reference data were subtracted from real-time images taken after perforant path fiber stimulation and were transferred at an interval of 0.6 ms. During each light flash, the DG was stimulated with a single voltage pulse (30 V for 400 μs) through a concentric bipolar electrode. Neuronal activity was detected as the ratio of the absorption signals due to neuronal excitation (ΔI) to the background light signals taken before stimulation of the DG (I_0_). One trial consisted of 512 sequential frames within 307 ms, and 16 trials were averaged to improve the signal-to-noise ratio. As a result, the background noise was reduced to 5% of the neuronal signal intensity. The fractional change in the optical signal was usually coded in a pseudocolor-scale. The temporal change of the optical signal was recorded from several unit areas on the spreading pathway of the brain slice to examine the time course of the optical signal. Each unit area corresponded to a 7x7 photodiode array (49 pixels) to improve the signal-to-noise ratio. The absorption signal by RH-482 reversed the polarity with an interference filter of 620 nm, which confirmed that the optical signal originated from RH-482 rather than from intrinsic signals or other sources [29]. The drugs were purchased from the following source: 6,7-dintroquinoxaline-2,3-dione (DNQX) and 2-Amino-5-phosphonopentanoic acid (APV) from Sigma-Aldrich (St. Louis, MO, USA).

### Preparation of dentate gyrus samples for FIB/SEM

Mice were anesthetized with sodium pentobarbital and then perfused with 4% paraformaldehyde and 0.125% glutaraldehyde in phosphate buffer (0.1 M, pH 7.4). Three to four hours after perfusion, the brains were removed, and coronal slices of the dorsal DG (300 μm) were cut with a vibrating blade microtome (VT1000S, Leica Microsystems, Nussloch, Germany). The slices were further fixed in a cacodylate buffer (0.1 M, pH 7.4) containing 2% paraformaldehyde and 2.5% glutaraldehyde for 6–12 hours at 4°C and were then washed with the same buffer. The slices were postfixed in a cacodylate buffer (0.1 M, pH 7.4) containing 2% OsO_4_/1.5% potassium ferrocyanide for 1 h on ice and were then washed five times with double distilled H_2_O (ddH_2_O). The slices were immersed in 1% thiocarbohydrazide for 1 h at 60°C. After being washed five times in ddH_2_O, the slices were then further immersed in 2% aqueous OsO_4_ for 1 hour on ice and were washed five times with ddH_2_O. The slices were then en bloc stained in a solution of 4% uranyl acetate dissolved in ddH_2_O overnight at 4°C and were washed five times with ddH_2_O. The slices were then further stained with Walton’s lead aspartate solution for 1 h at 60°C [30], dehydrated in an ethanol series (20%, 50% 70%, 90% and twice 100% for 10 min each), placed in ice-cold dry acetone for 10 min, subjected to infiltration of an epoxy resin (Epon 812, TAAB, England) mixture, and polymerized for 72 h at 60°C [25].

### Preparation of dentate gyrus samples with Golgi stain for FIB/SEM

Under deep anesthesia with sodium pentobarbital, the mice were perfused with phosphate buffer (0.1 M, pH 7.4) containing 4% paraformaldehyde and 1% glutaraldehyde and then with the same phosphate buffer containing 2% paraformaldehyde and 1% glutaraldehyde [31]. Three to four hours after perfusion, the brains were removed and processed using the Rapid GolgiStain kit according to manufacturer’s instructions (FD NeuroTechnologies, Columbia, MD, USA). The Golgi-stained slices of the DG (200 μm) were further processed with the protocol for FIB/SEM samples following en bloc staining.

### Imaging with FIB/SEM

The embedded slices were placed on a metal stub and further trimmed with glass and diamond knives in an ultramicrotome (Ultracut E microtome, Leica, Wetzlar, Germany). The slices were coated with a protective layer of carbon, which prevented any charge. The metal stub with the slices was set on the stage of FIB/SEM. The serial section images in the middle molecular layer of the dorsal DG were automatically obtained by FIB/SEM (Quanta 3D FEG, FEI, Hillsboro, OR, USA). Serial images of the block face were acquired by repeated cycles of sample surface milling and imaging using the Slice & View G2 operating software (FEI). The milling was performed with a gallium ion beam at 30 kV with a current of 1.0 nA or 3.0 nA (Golgi stain). The milling pitch was set to 15 or 30 (Golgi stain) nm/step. The images were acquired at an accelerating voltage of 2.5 kV. The other acquisition parameters were as follows: dwell time = 6 s/pixel, pixel size = 4.9 and 14.6 (Golgi stain) nm/pixel.

### Three-dimensional reconstruction and analysis

The serial section images were reconstructed to 3D images and were analyzed using Amira 5.4–5.5 software (FEI). The postsynaptic [e.g., dendritic spine and postsynaptic density (PSD)] and presynaptic (e.g., presynaptic bouton, synaptic vesicle and mitochondria) components were manually traced, and the volume of each component was measured using Amira. For the analyses of the postsynaptic components, three dendrites were traced from each mouse. The spines were classified into regular- and large-sized spines. A regular-sized spine was defined as having a spine volume less than or equal to the mean value + 2 standard deviations (SDs) of the volume obtained in the placebo groups (≤0.25 μm^3^), and a large-sized spine was defined as a spine with a volume greater than mean + 2SDs (>0.25 μm^3^). PSDs were identified as a band of electron-dense materials at asymmetrical synapses. For the analyses of the presynaptic components, the presynaptic boutons were classified by their connections to spines with different sizes (Group 1: spine volume < mean (0.076 μm^3^), Group 2: mean ≤ spine volume < mean + SD (0.162 μm^3^), Group 3: mean + SD ≤ spine volume < mean + 2 SDs (0.249 μm^3^), Group 4: mean + 2SDs ≤ spine volume) by using the mean and SD values from the placebo groups.

### Statistical analysis

The data are shown as the means ± SEM. Statistical analyses were performed using Student’s *t*-test, Mann-Whitney *U*-test, one-way ANOVA and two-way ANOVA (GraphPad Prism, GraphPad Software, San Diego, CA, USA), as indicated in figure legends.

## Results

### Neural activity of perforant path synapses in the DG after chronic fluoxetine treatment

**Fig 1:**
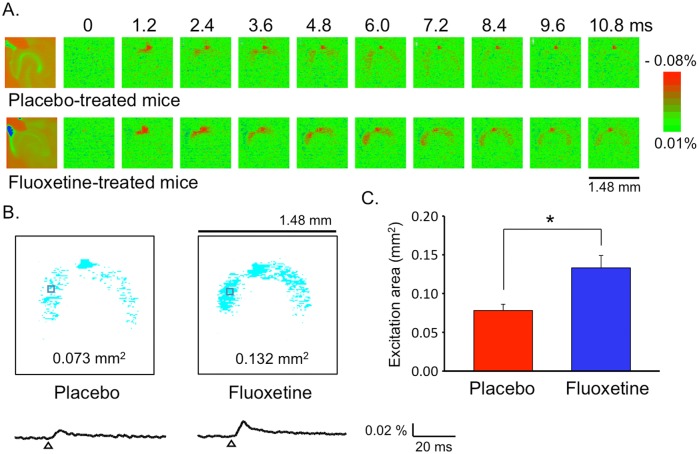
Effect of chronic fluoxetine treatment on optical responses in the hippocampal DG. (A) Effect of chronic fluoxetine treatment on optical responses evoked by the stimulation of the perforant path inputs in hippocampal slices. The left-most panel shows the pseudocolor image of the slice preparation in which the optical recordings were made. A series of optical images of neuronal activity were recorded at 1.2-ms intervals from 0 to 10.8 ms after nerve stimulation. The signal intensity, expressed as fractional changes in optical absorbance relative to the background (%), was coded by the pseudocolor image. (B, C) The maximum propagation area of the optical signal above the background noise was analyzed 7.2–9.6 ms after stimulation, when the activation of the dendrites of the GCs via glutamatergic synaptic transmission was detected (see S1 Fig). Typical images of the excitation area (B), traces of the optical responses at the boxed area (B) and the quantified excitation area (C) are shown in mice treated with placebo (n = 16 slices from 8 mice) and fluoxetine (n = 19 slices from 9 mice). *p < 0.001 compared with placebo-treated mice; Mann-Whitney *U*-test (U = 50, p = 0.0008). The preliminary data used for these figures are reported in a review article in Japanese [71].

The spatiotemporal propagation of neuronal excitation was recorded from neurons in the middle molecular layer of the hippocampal DG, where the perforant path inputs from layer II of the entorhinal cortex form synaptic connections with the dendritic spines of GCs (S1A–S1C Fig). The application of a single electrical stimulation (30 V for 400 μs) to the perforant path inputs in the middle molecular layer of the DG resulted in depolarizing optical responses adjacent to the stimulated site with a latency of 1.2 ms and their spreading in the middle molecular layer (Fig 1A and S1C Fig). The optical responses observed 7.2–9.6 ms after the electrical stimulation were considered to reflect the excitation of GC dendrites because the responses were blocked by AMPA and NMDA receptor antagonists (S1C and S1D Fig). The maximum propagation area of the optical signal observed 7.2–9.6 ms after nerve stimulation was determined by counting the number of pixels in which the evoked depolarizing optical signal was above the background noise. Pooled data showed that the maximum propagation area of the neuronal excitation was larger in the fluoxetine-treated mice than in the placebo-treated mice (Fig 1B and 1C). Analysis of the input-output relationship revealed that chronic fluoxetine treatment increased the amplitude of optical responses and the gain of the input-output curve (S2 Fig), although time course of the optical responses was not affected (Fig 1B and S2 Fig). Taken together, the efficacy of excitatory neurotransmission is enhanced in the perforant path-GC synapse in the fluoxetine-treated mice.

### Morphological changes in the spines of the perforant path-GC synapse in the DG after chronic fluoxetine treatment

**Fig 2:**
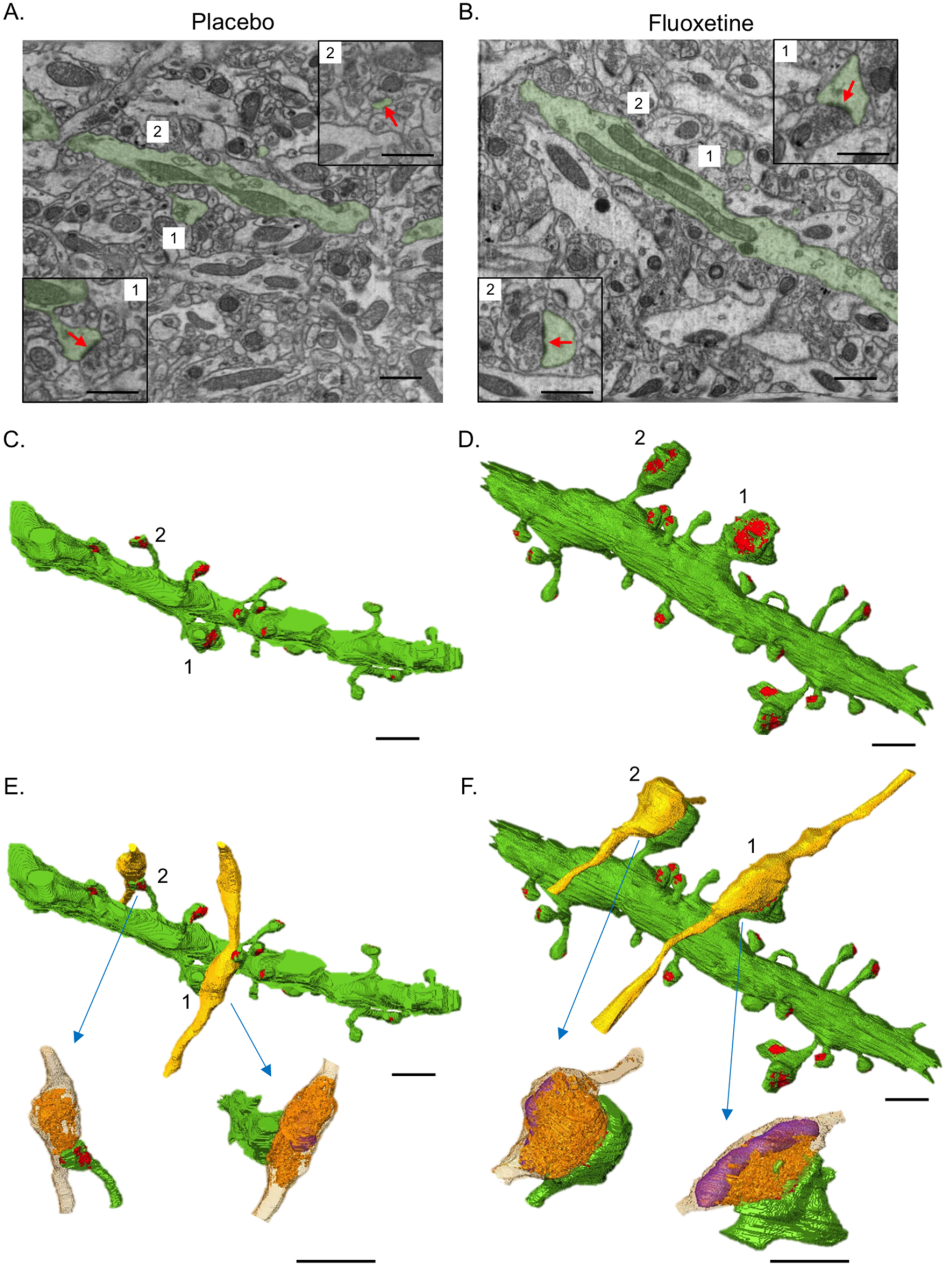
Three-dimensional reconstruction of perforant path-GC synapses in the dentate gyrus. (A, B) Full field SEM images obtained through FIB/SEM show the cross-sections of dendrites (green) in the placebo- (A) and fluoxetine (B)-treated mice. Insets show images of dendritic spines (green) and connecting boutons obtained in other sections. The arrows (red) indicate PSD. (C, D) 3D-reconstructed dendritic segments in the middle molecular layer of the DG in the placebo- (C) and fluoxetine (D)-treated mice. Note the appearance of the large-sized spines and PSDs (red) in the fluoxetine-treated mice. The dendritic spines, which are shown in the insets of Fig 1A and 1B, are indicated with numbers. (E, F) Three-dimensional reconstructed presynaptic boutons are visualized at two synapses in the placebo- (E) and fluoxetine (F)-treated mice. The synaptic vesicles (orange) and mitochondria (purple) are shown inside the presynaptic boutons. Note that the large-sized spines are in contact with large-sized presynaptic boutons. Scale bars: 1 μm.

We evaluated the effect of chronic fluoxetine treatment on the morphology of the perforant path-GC synapses in the middle molecular layer of the dorsal DG using serial images obtained with FIB/SEM (S3 Fig). Mice were treated with a fluoxetine pellet, which was designed to release fluoxetine at a constant rate of 15 mg/kg/day for 2 weeks. The 3D reconstruction of serial SEM images made it possible to visualize the EM-quality ultrastructure of the dendritic spines containing postsynaptic densities (PSDs) and the presynaptic boutons containing vesicles and mitochondria in the middle molecular layer of the DG (Fig 2). After chronic fluoxetine treatment, large-sized spines appeared in the dendritic spines of the GCs in the middle molecular layer. The large-sized spines contained large PSDs and formed contacts with the large boutons of the perforant path (Fig 2E and 2F).

**Fig 3:**
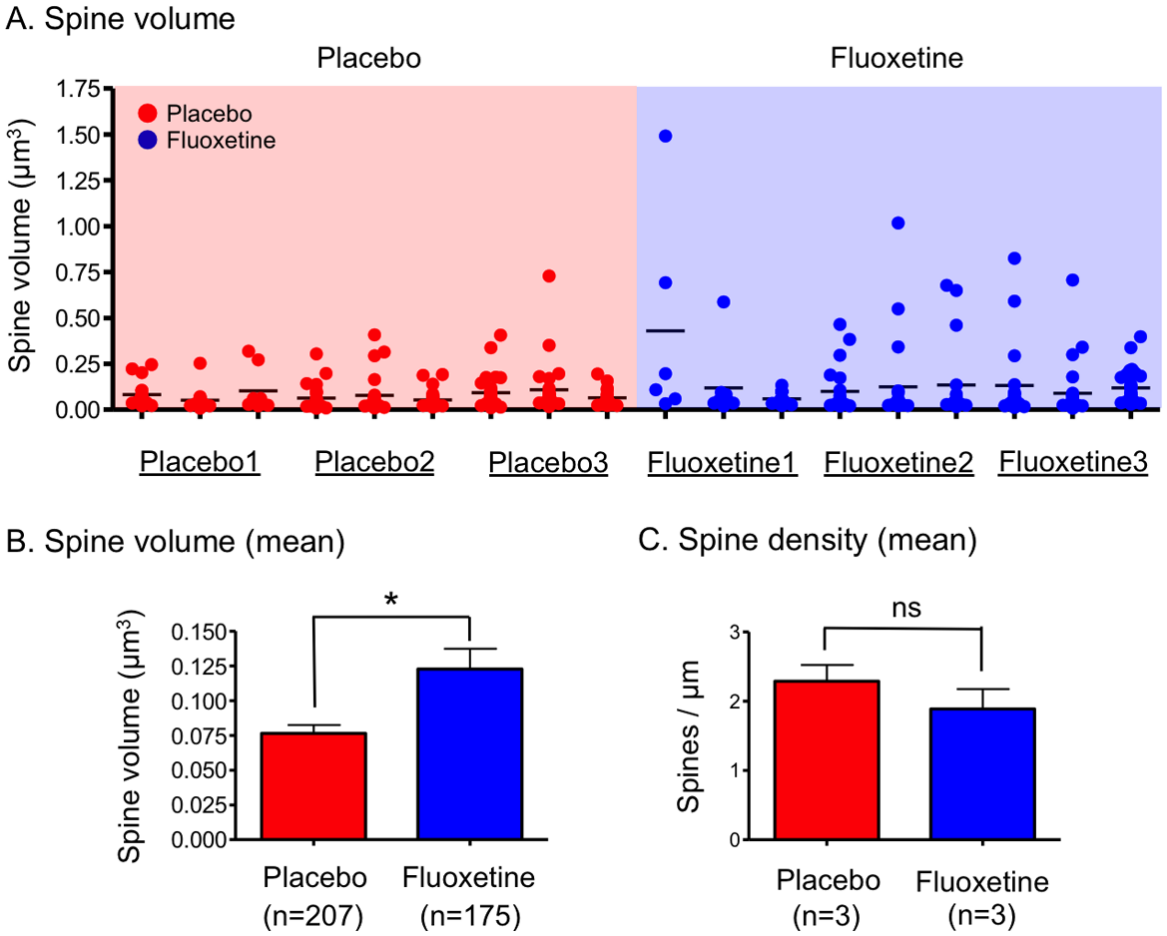
Spine volume and density in the placebo- and fluoxetine-treated mice. (A) The scatter plot shows the spine volume in mice treated with placebo (n = 207 spines from 9 dendrites, 3 dendrites per each of 3 mice) or fluoxetine (n = 175 spines from 9 dendrites, 3 dendrites per each of 3 mice). (B, C) Mean values of spine volume for all spines (Mann-Whitney *U*-test: U = 15930, p = 0.0425) (B) and spine density (Mann-Whitney *U*-test: U = 28, p = 0.2973) (C) in the placebo- or fluoxetine-treated mice. *p < 0.05 compared with the placebo-treated mice.

We then quantitatively analyzed the spine volume. The volume of each spine was quantitated using 3D reconstructed images of dendrites. Spines with a large volume were observed in all three fluoxetine-treated mice (Fig 3A). The mean value of the spine volume was significantly higher in the fluoxetine-treated mice than in the placebo-treated mice (Fig 3B), although the spine densities in the placebo- and fluoxetine-treated mice were similar (Fig 3C).

To classify the spines into regular- and large-sized spines, a large-sized spine was defined as a spine with a volume greater than or equal to the mean value + 2SDs of the placebo-treated mice (≥0.249 μm^3^). The percentages of large-sized spines were 5.3% and 11.4% in the placebo- and fluoxetine-treated mice, respectively. Histogram analyses of the spine volume revealed that the distribution of spine volumes of in regular-sized spines in the placebo- and fluoxetine-treated mice were similar (S4A and S4B Fig), but that, among large-sized spines, spines with extremely large volume (≥0.50 μm^3^) appeared after fluoxetine treatment (S4C Fig). In addition, the analysis of large-sized spines revealed multisynaptic spines that formed contacts with at lease two presynaptic boutons [32, 33] in three spines with volumes of 0.34, 0.65 and 1.02 μm^3^, and these were only found in the fluoxetine-treated mice (S5 Fig).

To avoid any bias in selection of dendrites with large-sized spines in the fluoxetine-treated mice, dendrites stained by chance with Golgi’s method were selected for the spine volume analysis (S6A Fig). All densely stained dendrites were subjected to 3D reconstruction, and the spine volume was quantitated. The 3D-reconstructed images of the Golgi-stained dendrites revealed the appearance of the large-sized spines after chronic fluoxetine treatment (S6A Fig). An increase in the spine volume in the Golgi-stained dendrites was observed in the fluoxetine-treated mice (S6B Fig). As expected, the spine density was not affected by the chronic fluoxetine treatment (S6C Fig). The results obtained from the Golgi-stained dendrites were consistent with those from manually selected dendrites (Fig 3).

### Effect of chronic fluoxetine treatment on PSDs of dendritic spines

**Fig 4:**
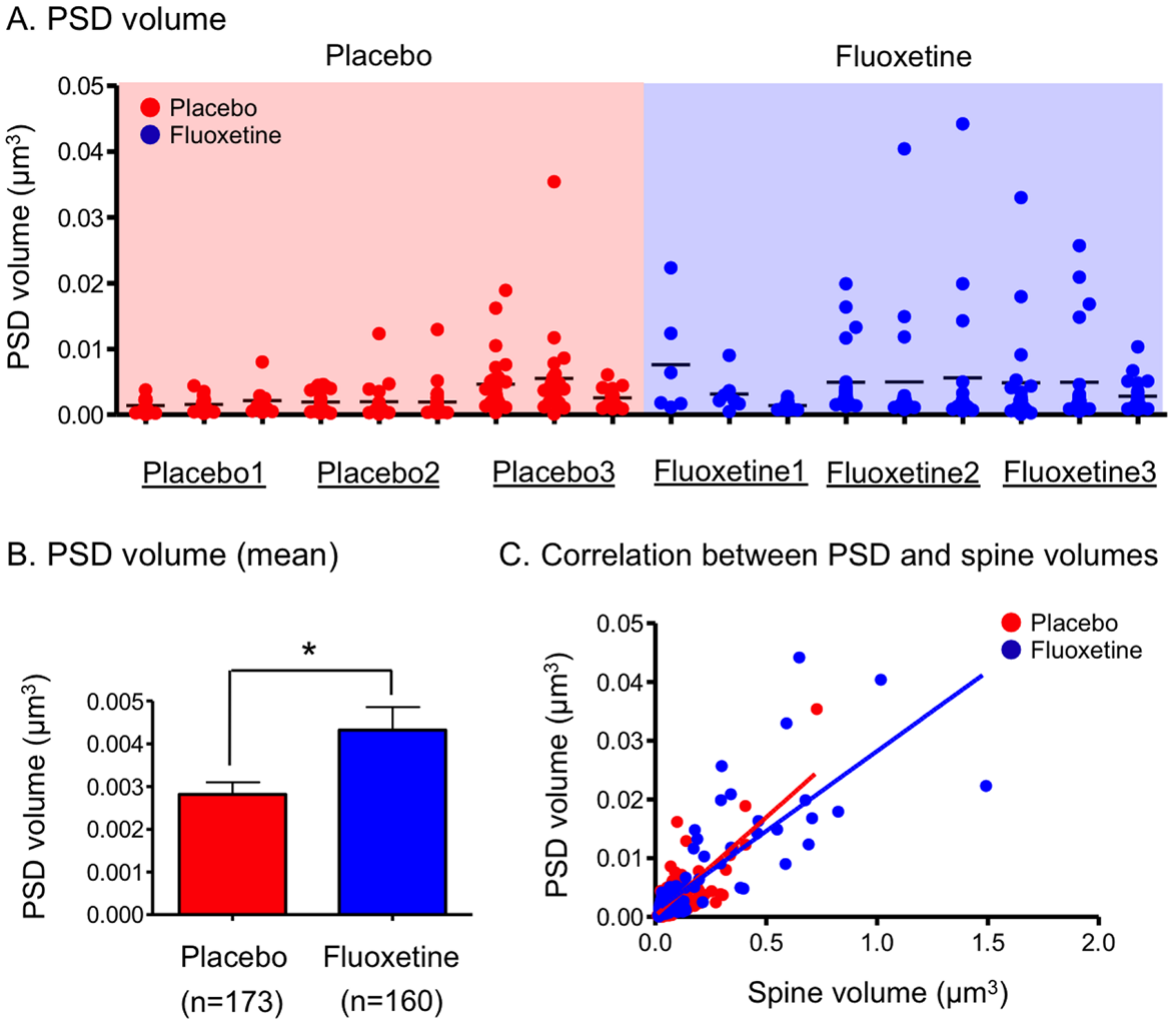
PSD volume and its correlation with spine volume. (A) Scatter plot showing the PSD volume in mice treated with placebo (n = 173 PSDs from 9 dendrites, 3 dendrites per each of 3 mice) or fluoxetine (n = 160 PSDs from 9 dendrites, 3 dendrites per each of 3 mice). (B) Mean values of the PSD volume in the placebo- and fluoxetine-treated mice. *p < 0.05 compared with placebo-treated mice; Mann-Whitney *U*-test (U = 11920, p = 0.0284). (C) Correlation between the PSD volume and spine volume in the placebo or fluoxetine-treated mice. The fitted lines for the mice treated with placebo (r^2^ = 0.63, *p* < 0.0001) or fluoxetine (r^2^ = 0.64, *p* < 0.0001) were obtained through a linear regression analysis.

We then evaluated the effect of chronic fluoxetine treatment on the PSD volume in each spine. PSDs with a large volume were mainly found in the fluoxetine-treated mice (Fig 4A). The mean PSD volume was significantly higher in the fluoxetine-treated mice than in the placebo-treated mice (Fig 4B). A significant correlation between PSD volume and spine volume was found in both the placebo- and fluoxetine-treated mice (Fig 4C), as had been previously reported [34]. However, this correlation was not affected by chronic fluoxetine treatment. When the spines were divided into regular and large-sized spines, a similar correlation between PSD and spine volumes was obtained in the regular-sized spines (S7A Fig). In the large-sized spines, the PSD volume did not correlate with the spine volume in the fluoxetine-treated mice (S7B Fig), suggesting that the PSD volume in the large-sized spines was already large and did not show an additional increase following the increase in spine volume.

### Effect of chronic fluoxetine treatment on presynaptic boutons in contact with spines of different sizes

**Fig 5:**
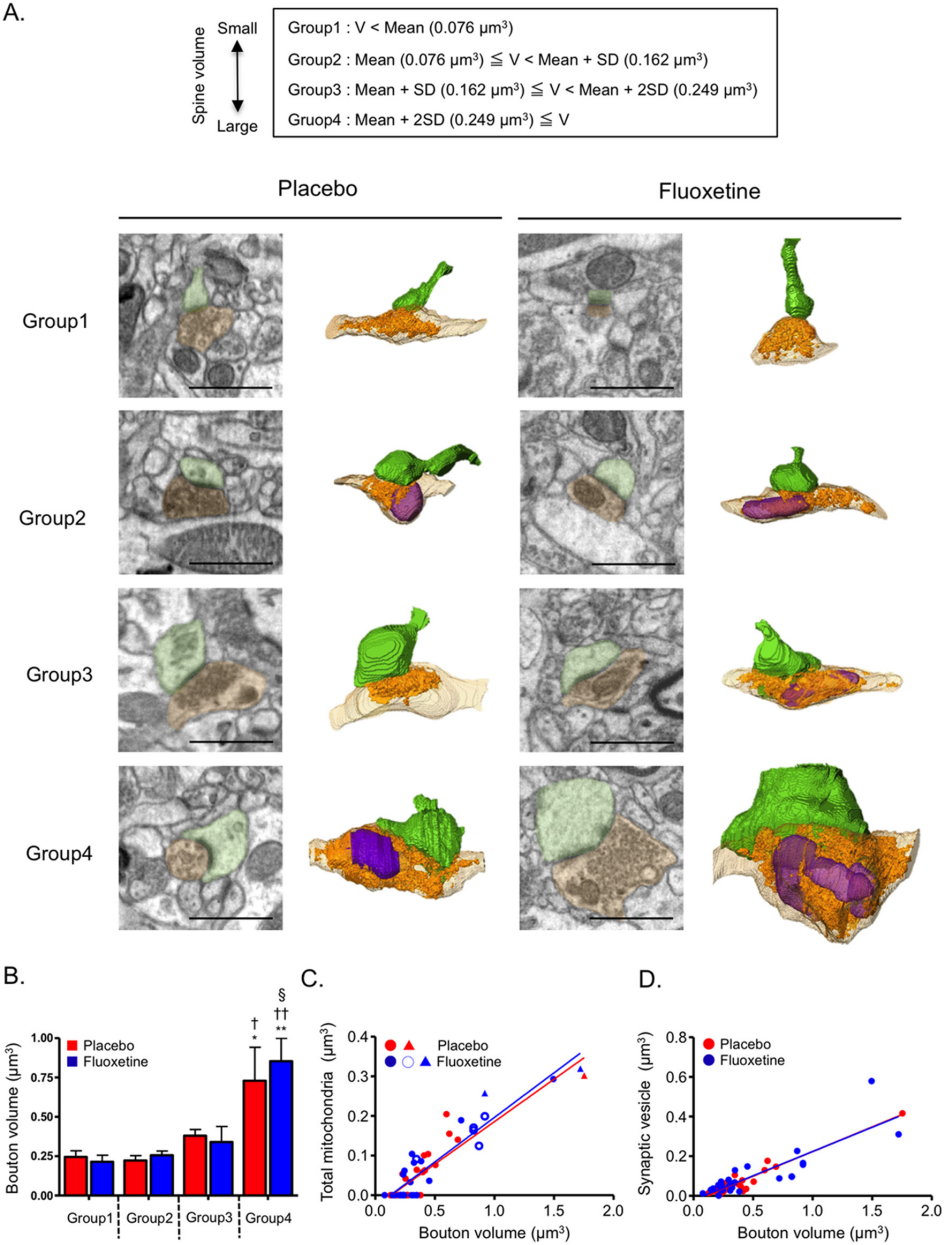
Characterization of presynaptic boutons connected to spines of different sizes. (A) Presynaptic boutons were classified by their connections to spines with different sizes. The spines were subdivided into four groups (Groups 1–4) based on the mean and SD values of the spine volume in placebo-treated mice. Typical images of SEM and 3D reconstructed spines (green) and connected boutons (light brown) are shown. Synaptic vesicles (orange) and mitochondria (purple) are shown inside the presynaptic boutons. Scale bars: 1 μm. (B) Volume of presynaptic boutons connected to spines classified as Groups 1–4. n = 6–10 presynaptic boutons in each group. No significant difference between placebo and fluoxetine with two-way ANOVA (drug effect, F_(1,44)_ = 0.0682, p < 0.794; group effect, F_(3,44)_ = 11.5, *p* < 0.0001; drug and group interaction, F_(3,44)_ = 0.230, *p* = 0.875). One-way ANOVA: placebo, F_(3,20)_ = 4.48, *p* = 0.0147; fluoxetine, F_(3,24)_ = 7.81, *p* = 0.0008; Bonferroni’s post-hoc test, *p < 0.05, compared with Group 1 in placebo, ^†^*p* < 0.05 compared with Group 2 in placebo, **p < 0.01 compared with Group 1 in fluoxetine, ^††^*p* < 0.01 compared with Group 2 in fluoxetine, ^§^*p* < 0.05 compared with Group 3 in fluoxetine. (C) Correlation between volumes of mitochondria and presynaptic boutons in mice treated with placebo (r^2^ = 0.79, *p* < 0.0001) or fluoxetine (r^2^ = 0.84, *p* < 0.0001) with linear regression analysis. The symbol indicates the number of mitochondrion in each presynaptic bouton: ●, one; ○, two; ▲, three. The mitochondrial volume is the sum of the volume for all of the mitochondria in each bouton. (D) Correlation between the volumes of synaptic vesicles and presynaptic boutons in mice treated with placebo (r^2^ = 0.91, *p* < 0.0001) or fluoxetine (r^2^ = 0.72, *p* < 0.0001) with linear regression analysis.

The volume of the presynaptic boutons in contact with spines of different sizes was evaluated. The spines were classified into four groups according to size (Groups 1–4) (Fig 5A, see Method). The regular-sized spines (spine volume < mean + 2SDs) were subdivided into three groups (Group 1–3), and the spines in Group 1–3 were in contact with boutons with similar volume in both the placebo- and fluoxetine-treated mice (Fig 5A and 5B). The large-sized spines, which were defined as Group 4 (mean + 2SDs ≤ spine volume), were in contact with boutons with larger volume than those in Group 1, 2 or 3 in both groups of mice (Fig 5A and 5B). The relationship between spine and bouton volumes was not affected by chronic fluoxetine treatment.

It is well known that the volumes of the mitochondria and synaptic vesicles in presynaptic boutons are associated with synaptic activity [35, 36]. We therefore examined whether the volume of mitochondria or synaptic vesicles correlates with the volume of the presynaptic boutons. The total mitochondria volume was positively correlated with the presynaptic bouton volume (Fig 5C), and similar correlations were found in the placebo- and fluoxetine-treated mice. In the boutons in contact with the Group 4 spines, 60% of spines (6/10 spines) in the fluoxetine-treated mice, but only 16.7% of spines (1/6 spines) in the placebo-treated mice, contained two or three mitochondria. However, the size of each mitochondrion was similar despite the number of mitochondria in a presynaptic bouton (data not shown). Furthermore, the volume of all of the synaptic vesicles in each bouton correlated with the volume of the boutons (Fig 5D). These results suggested that the presynaptic bouton in contact with the large-sized spine was large in volume and contained mitochondria with a large volume and a large amount of synaptic vesicles.

## Discussion

The imaging of neural tissues using FIB/SEM and 3D reconstruction of the synapse from serial EM images is a powerful technique for analyzing the fine structures of synaptic components. Using this technique, the present study demonstrated that chronic fluoxetine treatment induced the appearance of large-sized perforant path-GC synapses without affecting spine density in the middle molecular layer of the DG, where excitatory synaptic transmission was expected to be enhanced after chronic fluoxetine, as found with voltage-sensitive dye imaging and reported previously [12–14]. At the perforant path-GC synapses with large-sized spines, the large-sized spines were connected to large-sized boutons. The large-sized spines contained large-sized PSDs, and the large-sized boutons contained synaptic vesicles and mitochondria with large sizes in correlation with the bouton volume. Thus, large-sized perforant path-GC synapses appeared after chronic fluoxetine treatment and had pre- and post-synaptic components required for excitatory synaptic transmission, suggesting that the enlarged perforant path-GC synapse may contribute to chronic fluoxetine treatment-induced synaptic plasticity, resulting in enhanced excitatory synaptic transmission.

### Alterations of spine morphology after chronic fluoxetine treatment

Chronic fluoxetine treatment induced the appearance of large-sized spines in the middle molecular layer of the DG. Several studies have previously demonstrated that chronic fluoxetine treatment induced synaptic remodeling, such as changes in spine density and size in the hippocampus. In ovariectomized female rats, chronic fluoxetine (5 mg/kg/day, i.p. for 5–14 days) has been shown to increase the density of spine synapses on pyramidal cells in the stratum radiatum of the CA1 and CA3 regions as determined through images from a transmission electron microscope (TEM) [19]. In normal male mice, chronic fluoxetine treatment (16 mg/kg/day in drinking water for 28 days) also increased the spine density along the apical dendrites of the pyramidal cells in the CA1 region, as determined through an analysis of Golgi-stained images [20]. Regarding spine morphology, chronic fluoxetine treatment (0.7 mg/kg/day, i.p. for 28 days) is shown to increase mature, mushroom-type spines as well as spine density at the Schaffer collateral-CA1 synapse, but not at the perforant path-CA1 synapse, in Golgi-stained images [37]. McAvoy et al. reported that the hippocampus of middle-aged female mice (10 months of age) was more sensitive to fluoxetine-induced synaptic remodeling than the hippocampus of adult female mice (4 months of age), as demonstrated through an analysis of fluorescent images in Thy1-GFP mice, in which mature granule cells and CA1 pyramidal cells were genetically labeled with GFP [21]. In the outer molecular layer of the DG, chronic fluoxetine treatment (18 mg/kg/day in drinking water for 28 days) induced an increase in spine density and a shift of the spine head diameter distribution toward larger spines in mature granule cells in middle-aged mice but not adult mice [21].

Chronic fluoxetine treatment-induced increases in spine density have been detected in several regions of the hippocampus (DG, CA3 and CA1) under various experimental conditions (e.g., mouse and rat, male and female, age, dose and duration of fluoxetine, imaging method) [19–21]. However, an increase in spine density after chronic fluoxetine treatment was not observed in this study. It is possible that the remodeling of the spine density is less sensitive to fluoxetine in the middle molecular layer of the DG compared to the outer molecular layer of the DG or other hippocampal areas [21]. Regarding the spine size, one study [21] evaluated the effect of fluoxetine on the spine size in the hippocampus using fluorescent images. Our study constitutes the first morphological analysis using EM-quality images. The 3D reconstruction of spine structure using FIB/SEM images is a sensitive technique for detecting changes in spine morphology and therefore allowed the detection of the chronic fluoxetine treatment-induced appearance of large-sized spines in the middle molecular layer of the DG in adult mice.

Chronic fluoxetine treatment has been shown to enhance adult neurogenesis in the DG [5, 6]. After chronic fluoxetine treatment, a larger number of newborn granule cells can be integrated into the hippocampal circuitry compared with that observed under control conditions. Newborn granule cells within 4 weeks are estimated to equal 6% of the total granule cells in the rat DG [38]. Even taking into account the fact that chronic fluoxetine treatment increases proliferating cells by 40–60% in the subgranular zone of the DG [5, 6, 39], the majority (>90%) of GCs consist of mature neurons. As large-sized spines were detected in eight out of nine dendrites in the fluoxetine-treated mice (see Fig 3A), the possibility that the detection of large-sized spines after chronic fluoxetine treatment was due to the replacement of GCs with newly integrated neurons is very low. Taken together, the data show that chronic fluoxetine treatment likely induces changes in spine morphology in the preexisting mature GCs.

Mechanisms for spine enlargement have been investigated intensively under various experimental conditions [15, 40]. BDNF/TrkB signaling plays a critical role in spine enlargement associated with synaptic plasticity [41, 42]. In fact, chronic fluoxetine treatment increases the BDNF levels in the hippocampus [43], and an increase in the BDNF level in the DG was observed in our experimental conditions (Kuroiwa M and Shuto T, unpublished observation), suggesting that the upregulation of BDNF/TrkB signaling may have contributed to the fluoxetine-induced enlargement of the spines. However, this up-regulation of BDNF/TrkB signaling and the subsequent changes in spine morphology likely occurred at the selected spines and not evenly at all of the spines. The selection of spines could have been due to the high excitability of the selected spines [44, 45] or to the connected perforant path boutons [46, 47], although this suggestion is highly speculative.

### Enlarged presynaptic boutons connected to large-sized spines after chronic fluoxetine treatment

Large-sized spines induced by chronic fluoxetine treatment were connected to boutons with a large size, and the correlation of the spine and bouton volumes was maintained after chronic fluoxetine treatment. The function and/or morphology of presynaptic boutons is regulated by neural activity [48], cell adhesion molecules [49], and secreted factors [50], including BDNF [51, 52], Wnt7 [53, 54] and fibroblast growth factor 22 (FGF22) [55, 56]. Antidepressants are known to upregulate secreted factors (*e*.*g*., BDNF, VEGF, FGF and Wnt) and their downstream signaling [57], and these factors, most notably BDNF, are expected to contribute to the enlargement of the boutons after chronic fluoxetine treatment.

In general, neurons with high activity have large boutons compared with neurons with low activity [56]. However, Schikorski et al. [58, 59] indicated that bouton sizes in various types of neurons show a 10-fold difference. Even along single axons, boutons are heterogeneous, and their sizes are variable [60]. These findings suggest that neural activity may be one of the factors that regulate the size of the boutons, but many other factors regulate bouton morphology. Fluoxetine-induced plasticity at the perforant path-GC synapses can be attributable to changes in neural activity at the presynaptic boutons [14] as well as at the postsynaptic spines [12, 14]. Wang et al. [14] reported that chronic fluoxetine treatment induced a reduction of paired pulse depression at the perforant path-GC synapses. It is possible that similar mechanisms with the presynaptic plasticity may be involved in bouton enlargement. The precise mechanisms for bouton enlargement after chronic fluoxetine need to be clarified.

### Function of fluoxetine-induced large-sized synapses

#### Postsynaptic spine function

It has been reported that spine size is correlated with PSD size and synaptic strength [34]. Meyer et al. [46] recently reported that synaptic plasticity induced by glutamate uncaging accompanies a balanced enlargement of the spines, PSD and boutons in a CA1 slice culture, suggesting that the correlated enlargement of the synaptic components is essential for the enhancement of synaptic strength. In our study, the fluoxetine-induced large-sized spine contained large-sized PSDs. PSDs are correlated with spines in terms of volumes when analyzed with all- or regular-sized spines, but this correlation was lost in the fluoxetine-induced large-sized spines. Because the PSD volume in the large-sized spines was markedly larger than that in the regular-sized spines, the increase in PSD volume in the large-sized spines may have already saturated the potential to further increase the PSD volume. In addition, the fluoxetine-induced large-sized spines contained the spine apparatus (data not shown) [61], and 15% of them were connected to multiple boutons (multisynaptic spine) [32, 33]. These findings suggest that the large-sized spines may be associated with increased synaptic transmission.

Chronic treatment with antidepressants has been considered to facilitate neural plasticity [62, 63]. However, some studies failed to detect the facilitation of LTP by chronic fluoxetine treatment [37, 64]. Rubio et al. [37] reported that chronic fluoxetine induced deficits in LTP and LTD at the Schaffer collateral-CA1 synapse. The deficits in LTP and LTD seem to be associated with the increases in spine density and mushroom-type spines, because LTP or LTD was not affected in the perforant path-CA1 synapse where spine morphology was unaltered. Furthermore, fluoxetine-induced mushroom-type spines have been shown to recruit Ca^2+^-impermeable, GluA2-containing AMPA receptors at PSD in the cerebral cortex [65], suggesting that the decrease in Ca^2+^ permeability via AMPA receptors may result in deficits in synaptic plasticity [37]. Thus, fluoxetine induces alteration of synaptic plasticity by affecting molecular components, and such factors should be taken into account for functional interpretation.

#### Presynaptic bouton function

The mitochondria and synaptic vesicles inside the large-sized boutons were large in correlation with the bouton volume. The findings are consistent with previous reports showing the correlation of mitochondria [66] or synaptic vesicles [67, 68] with bouton volume. Prior evidence that the mitochondria in the boutons enhance synaptic vesicle release [35] and that the increased amounts of synaptic vesicles are related to the increased synaptic transmission [69] suggest that the enlarged presynaptic boutons have an increased ability of excitatory synaptic transmission.

The large-sized perforant path-GC synapses induced by chronic fluoxetine consist of presynaptic boutons and postsynaptic spines with large components that facilitate the excitatory synaptic transmission. The facilitation of excitatory inputs to the hippocampus may play a critical role in adjustment of hippocampal networks in response to antidepressant medication, leading to gradual improvements in disturbed information processing within affected neural networks [70].

## Conclusions

The 3D reconstruction of perforant path-GC synapses using FIB/SEM images allowed successfully visualization of the complex structures of the pre- and post-synaptic components. Three-dimensional images with quantitative data revealed that chronic fluoxetine treatment resulted in the appearance of large-sized synapses, which may be functionally related to enhanced synaptic transmission.

## Supporting Information

S1 Fig Optical responses in the DG evoked by stimulation of the perforant path inputs. (A, B) A pseudocolor image of the slice preparation in which optical recordings were made (A) and a schematic illustration of a hippocampal slice (B). The position of electrical stimulation (30 V for 400 μs) in the middle molecular layer of the DG is indicated. (C) Optical responses evoked by electrical stimulation in a hippocampal slice. A series of optical images of neuronal activity was recorded at 1.2-ms intervals from 0 to 10.8 ms after nerve stimulation in the absence (-) or presence (+) of an AMPA receptor antagonist, DNQX (20 μM), and an NMDA receptor antagonist, APV (40 μM). The time after stimulation is indicated at the top of each panel. All records were taken from the same slice. The signal intensity, expressed as the fractional change in optical absorbance relative to the background (%), was coded based on a pseudocolor scale. (D) The amplitude of the optical signal after stimulation was recorded at three positions (#1, #2, #3) located at different distances from the stimulatory electrode. The arrowheads indicate the time of stimulation. At position of #3 (500 μm from the stimulation electrode), optical responses were completely blocked by DNQX plus APV. The ordinate and abscissa scale bars indicate the fractional change in light intensity and time, respectively.
(TIF)

S2 Fig Input-output relationship of the optical responses. (A) Traces of the optical responses evoked by stimulus intensities of 6, 10 and 30 V at position #3 (S1D Fig) in hippocampal slices. (B) The input-output relationship of the optical responses in mice treated with placebo (n = 10 slices from 5 mice) and fluoxetine (n = 12 slices from 6 mice). Signal intensity is expressed as the fractional change in optical absorbance relative to the background (%). Data represents means ± SEM. Two-way repeated measures ANOVA: drug effect, F_(1,100)_ = 5.090, p < 0.0354; time effect, F_(5,100)_ = 109.1, *p* < 0.0001; drug and time interaction, F_(5,100)_ = 2.661, *p* = 0.0266); Bonferroni’s post-hoc test: *p < 0.05 compared with placebo.
(TIF)

S3 Fig Analysis area of the middle molecular layer of the dorsal DG for FIB/SEM imaging. (A) Schematic view of the FIB/SEM apparatus and the stained hippocampal slice embedded in epoxy resin. Serial sample surface milling was performed with a focused Ga ion beam (FIB), and each surface was imaged by SEM. (B, C) Analysis area in the middle molecular layer (MML) of the dorsal DG at low (80x) (B) and high (250x) (C) magnification. At the analysis area (black rectangle), serial SEM images were obtained by repeated surface milling with FIB. OML, outer molecular layer; IML, inner molecular layer; GCL, granule cell layer; SGZ, subgranular zone.
(TIF)

S4 Fig Distribution of spine volume in regular and large-sized spines in the placebo- and fluoxetine-treated mice. Histogram analyses of the spine volume of all spines (A), regular-sized spines (B) and large-sized spines (C) in the placebo- or fluoxetine-treated mice. The large-sized spine was defined as the spine with a volume greater than or equal to the mean value + 2SDs of the placebo-treated mice (≥ 0.249 μm^3^). Green and yellow boxes indicate the regular- and large-sized spines, respectively.
(TIF)

S5 Fig Multisynaptic spines in the fluoxetine-treated mice. (A) A field SEM image showing the cross-section of a multisynaptic spine and a 3D reconstructed image of a multisynaptic spine in a fluoxetine-treated mouse. The multisynaptic spine connected to two axons, axon 1 (red) and axon 2 (blue). (B, C) Tables show the percentage of multisynaptic spines in large-sized spines (B) and the volume of multisynaptic spines and number of connected axons (C).
(TIF)

S6 Fig Analysis of spine volume in the Golgi-stained dendrites. (A) Three-dimensional reconstruction of dendritic spines stained with the Golgi’s method using FIB/SEM. Golgi staining was performed in two placebo- and two fluoxetine-treated group. All of the following stained dendrites in the serial SEM images were analyzed: placebo 1 (n = 311 spines/4 dendrites), placebo 2 (n = 922 spines/13 dendrites), fluoxetine 1 (n = 138 spines/2 dendrites) and fluoxetine 2 (n = 260 spines/4 dendrites). (B, C) Mean spine volume and density in the placebo- or fluoxetine-treated mice.
(TIF)

S7 Fig Correlation between the PSD volume and spine volume in the regular and large-sized spines. (A) In the regular-sized spines, similar correlations between the PSD and spine volumes were obtained in the placebo- and fluoxetine-treated mice (Placebo: r^2^ = 0.28, *p* < 0.0001; Fluoxetine: r^2^ = 0.44, *p* < 0.0001) with linear regression analysis. (B) In the large-sized spines of the fluoxetine-treated mice, the PSD volumes were not significantly correlated with the spine volume (r^2^ = 0.13, *p* = 0.118). In the placebo-treated mice, a linear regression analysis was not performed because most of the spine volume was distributed in a narrow range (0.249–0.500).
(TIF)
