# Supplementary figures and images for: Chronic Fluoxetine Induces the Enlargement of Perforant Path-Granule Cell Synapses in the Mouse Dentate Gyrus
Source: PLoS One. 2016 Jan 20;11(1):e0147307. doi: 10.1371/journal.pone.0147307 (PMC4720354; doi:10.1371/journal.pone.0147307)

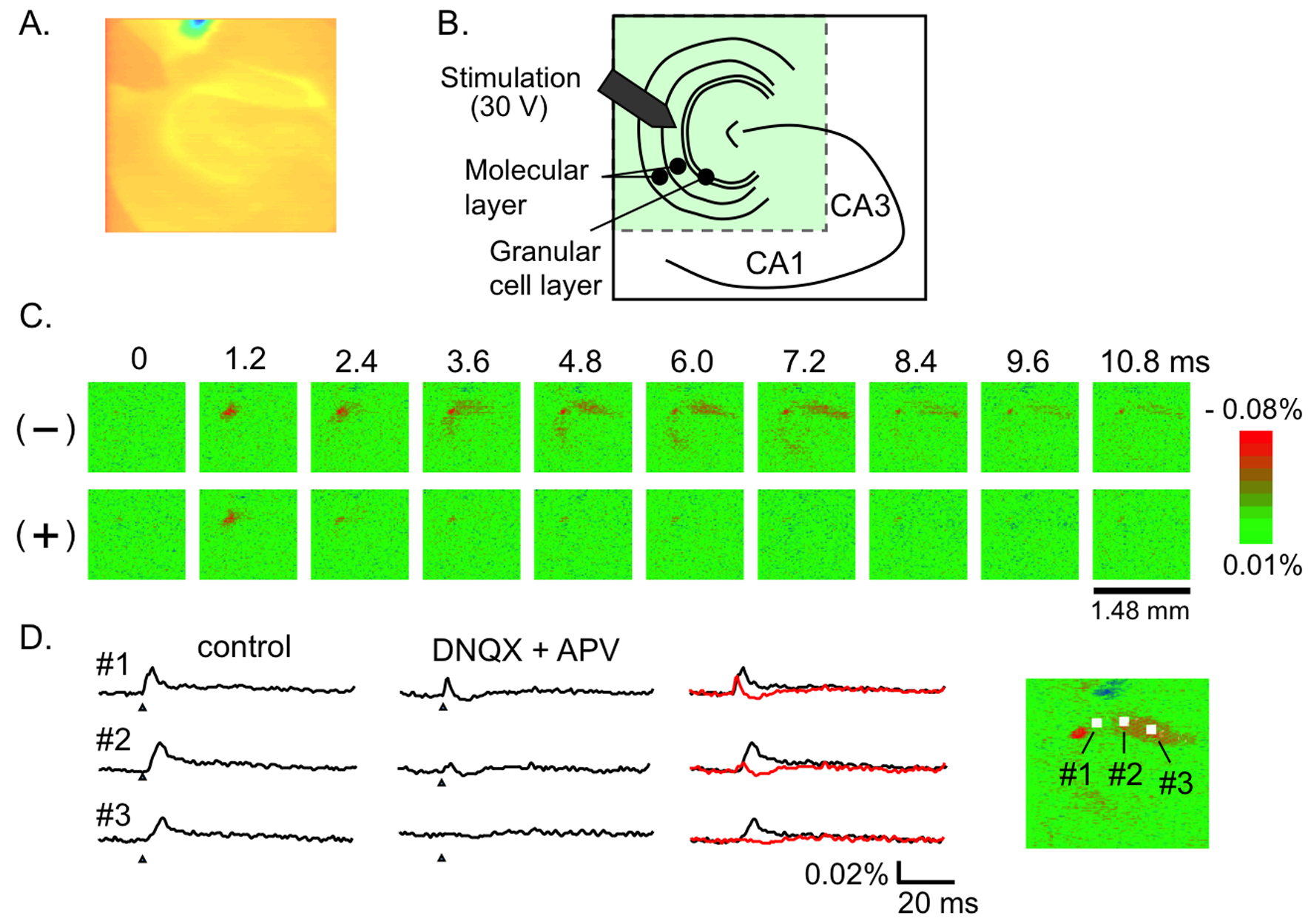

Supplement: S1 Fig — (A, B) A pseudocolor image of the slice preparation in which optical recordings were made (A) and a schematic illustration of a hippocampal slice (B). The position of electrical stimulation (30 V for 400 μs) in the middle molecular layer of the DG is indicated. (C) Optical responses evoked by electrical stimulation in a hippocampal slice. A series of optical images of neuronal activity was recorded at 1.2-ms intervals from 0 to 10.8 ms after nerve stimulation in the absence (-) or presence (+) of an AMPA receptor antagonist, DNQX (20 μM), and an NMDA receptor antagonist, APV (40 μM). The time after stimulation is indicated at the top of each panel. All records were taken from the same slice. The signal intensity, expressed as the fractional change in optical absorbance relative to the background (%), was coded based on a pseudocolor scale. (D) The amplitude of the optical signal after stimulation was recorded at three positions (#1, #2, #3) located at different distances from the stimulatory electrode. The arrowheads indicate the time of stimulation. At position of #3 (500 μm from the stimulation electrode), optical responses were completely blocked by DNQX plus APV. The ordinate and abscissa scale bars indicate the fractional change in light intensity and time, respectively. (TIF) [file pone.0147307.s001.tif]

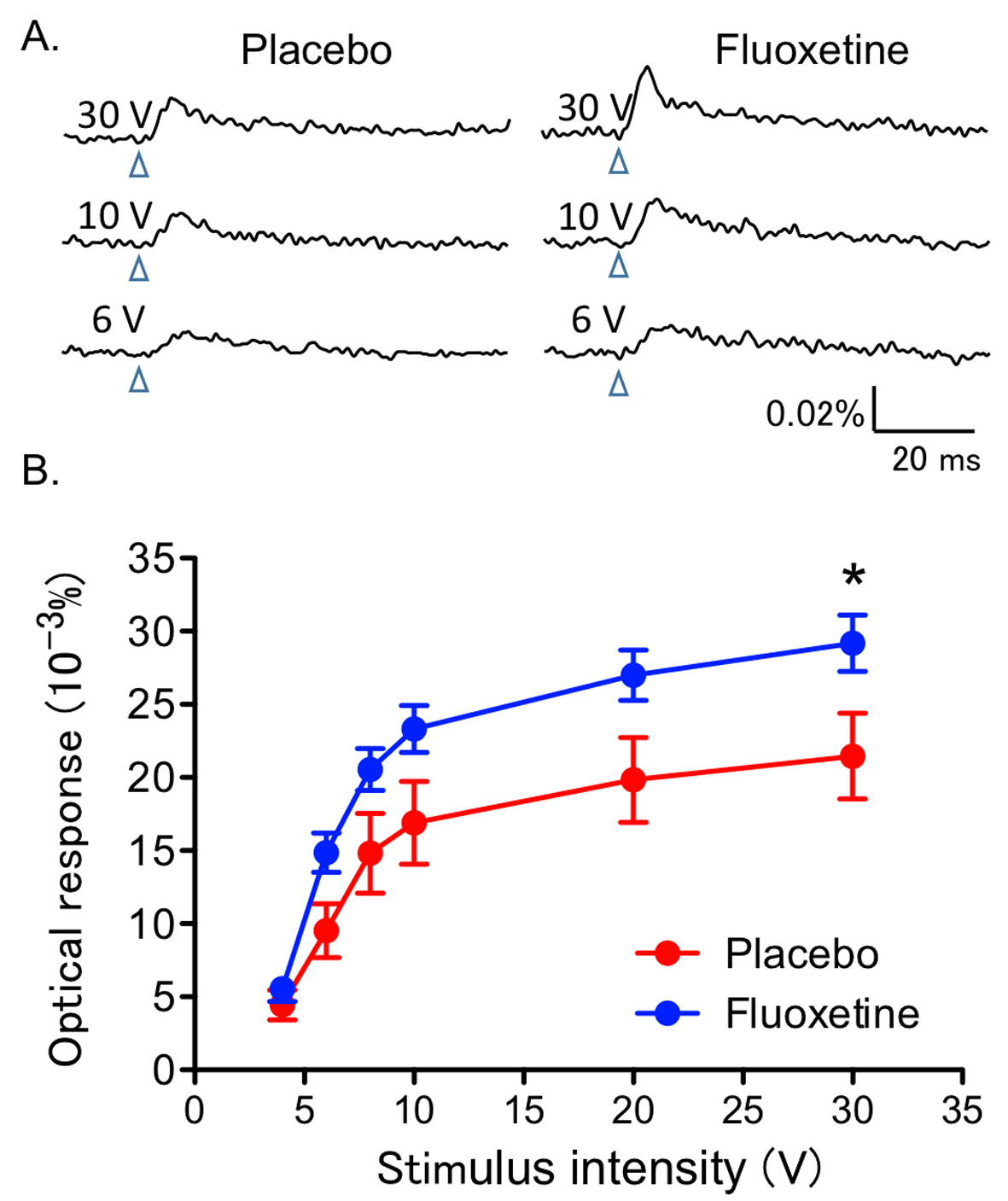

Supplement: S2 Fig — (A) Traces of the optical responses evoked by stimulus intensities of 6, 10 and 30 V at position #3 (S1D Fig) in hippocampal slices. (B) The input-output relationship of the optical responses in mice treated with placebo (n = 10 slices from 5 mice) and fluoxetine (n = 12 slices from 6 mice). Signal intensity is expressed as the fractional change in optical absorbance relative to the background (%). Data represents means ± SEM. Two-way repeated measures ANOVA: drug effect, F(1,100) = 5.090, p < 0.0354; time effect, F(5,100) = 109.1, p < 0.0001; drug and time interaction, F(5,100) = 2.661, p = 0.0266); Bonferroni’s post-hoc test: *p < 0.05 compared with placebo. (TIF) [file pone.0147307.s002.tif]

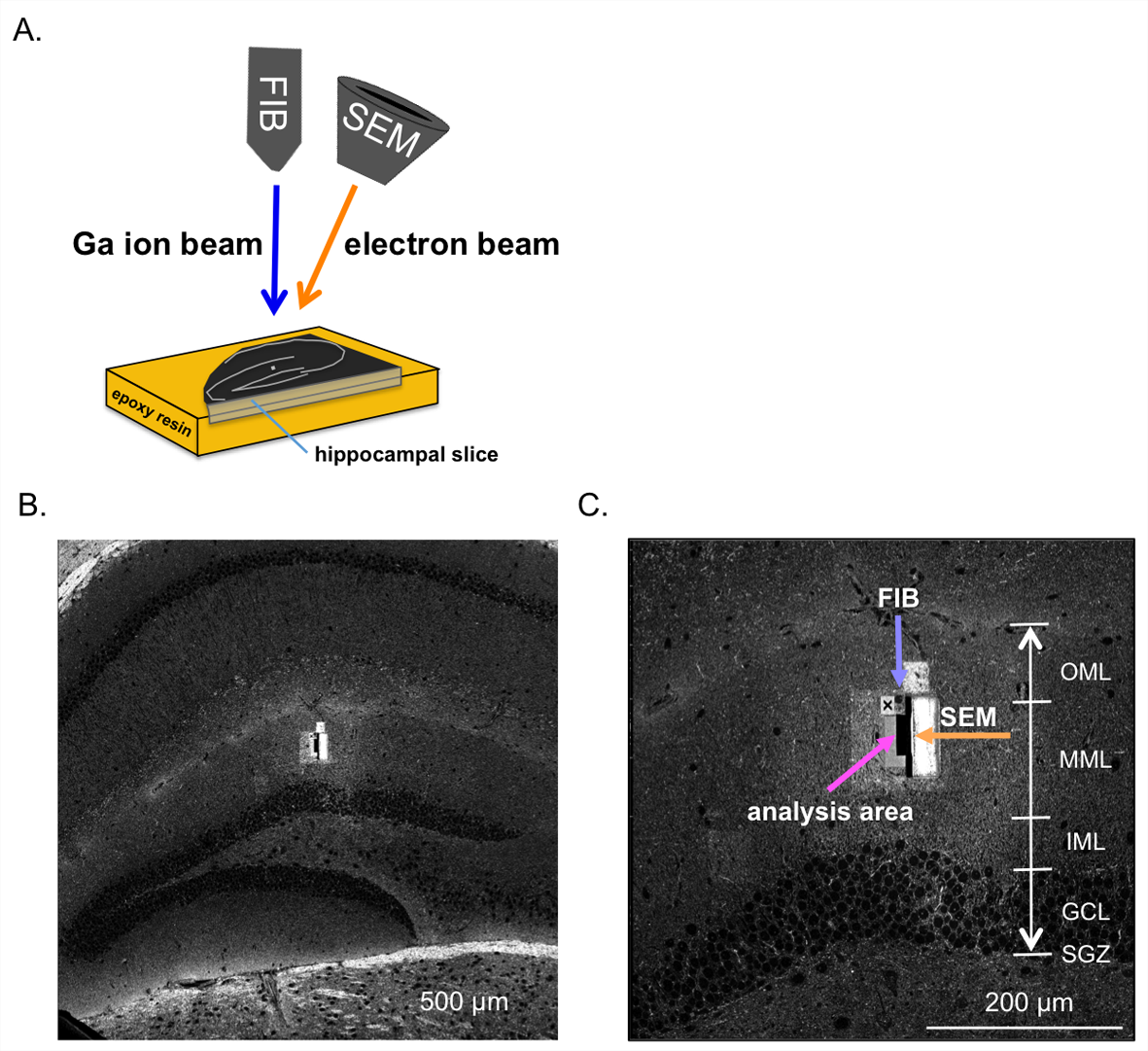

Supplement: S3 Fig — (A) Schematic view of the FIB/SEM apparatus and the stained hippocampal slice embedded in epoxy resin. Serial sample surface milling was performed with a focused Ga ion beam (FIB), and each surface was imaged by SEM. (B, C) Analysis area in the middle molecular layer (MML) of the dorsal DG at low (80x) (B) and high (250x) (C) magnification. At the analysis area (black rectangle), serial SEM images were obtained by repeated surface milling with FIB. OML, outer molecular layer; IML, inner molecular layer; GCL, granule cell layer; SGZ, subgranular zone. (TIF) [file pone.0147307.s003.tif]

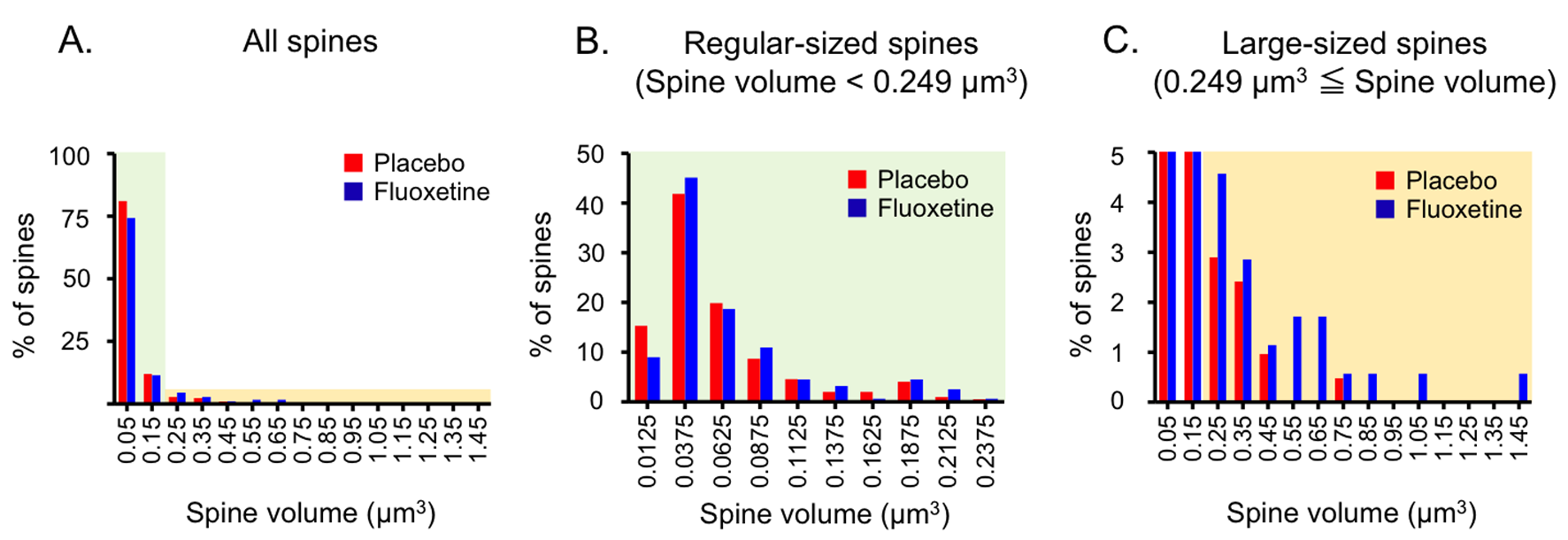

Supplement: S4 Fig — Histogram analyses of the spine volume of all spines (A), regular-sized spines (B) and large-sized spines (C) in the placebo- or fluoxetine-treated mice. The large-sized spine was defined as the spine with a volume greater than or equal to the mean value + 2SDs of the placebo-treated mice (≥ 0.249 μm3). Green and yellow boxes indicate the regular- and large-sized spines, respectively. (TIF) [file pone.0147307.s004.tif]

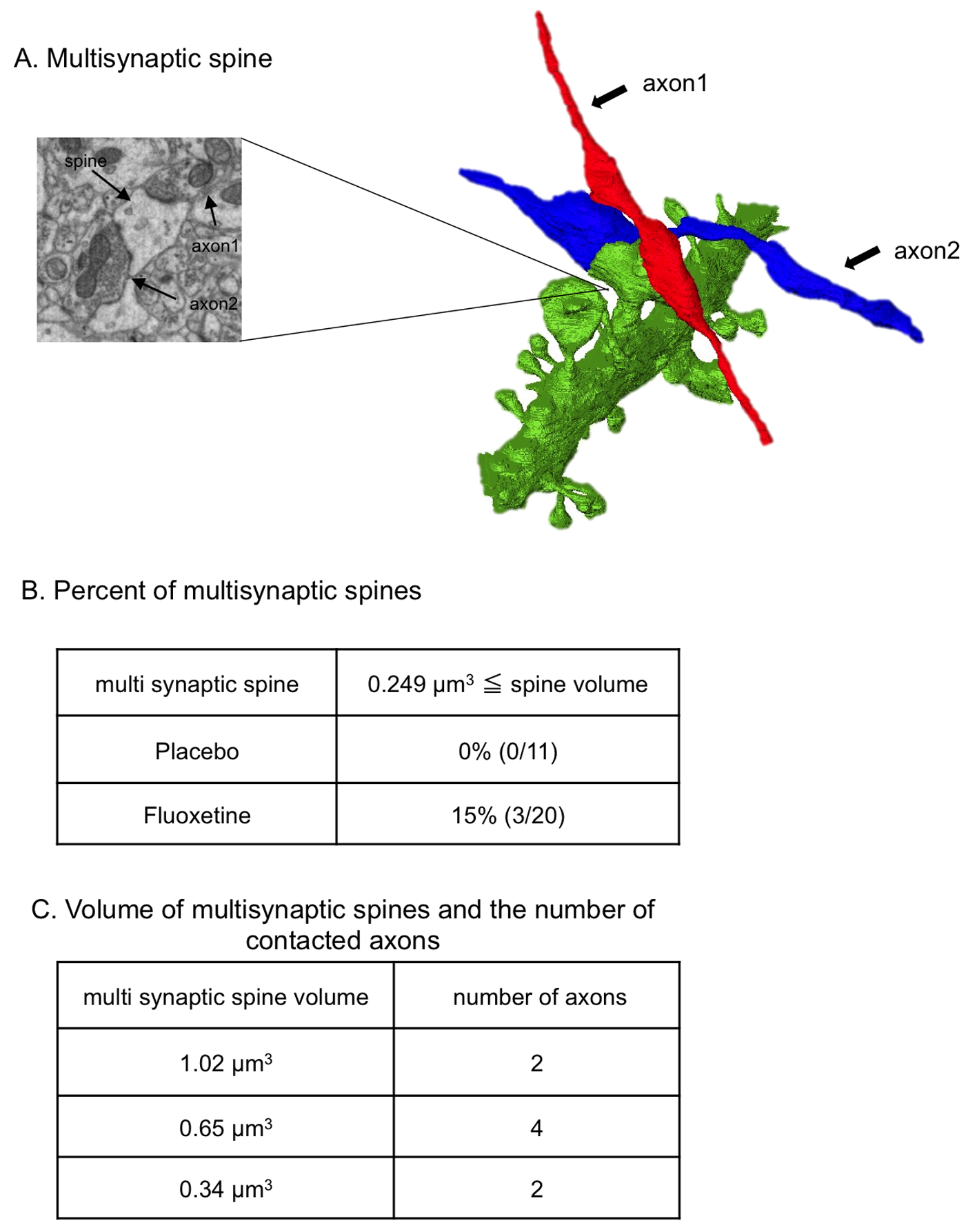

Supplement: S5 Fig — (A) A field SEM image showing the cross-section of a multisynaptic spine and a 3D reconstructed image of a multisynaptic spine in a fluoxetine-treated mouse. The multisynaptic spine connected to two axons, axon 1 (red) and axon 2 (blue). (B, C) Tables show the percentage of multisynaptic spines in large-sized spines (B) and the volume of multisynaptic spines and number of connected axons (C). (TIF) [file pone.0147307.s005.tif]

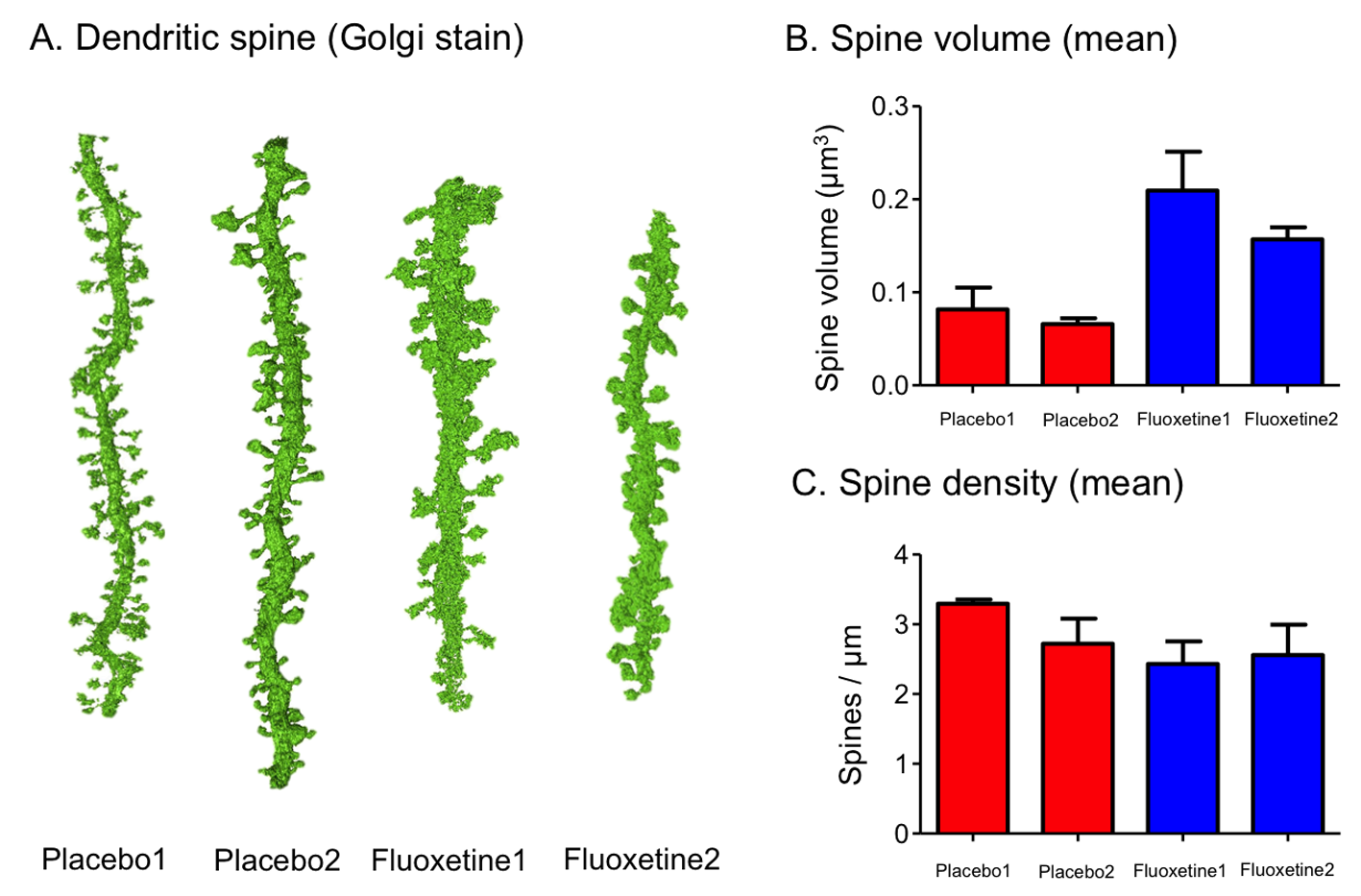

Supplement: S6 Fig — (A) Three-dimensional reconstruction of dendritic spines stained with the Golgi’s method using FIB/SEM. Golgi staining was performed in two placebo- and two fluoxetine-treated group. All of the following stained dendrites in the serial SEM images were analyzed: placebo 1 (n = 311 spines/4 dendrites), placebo 2 (n = 922 spines/13 dendrites), fluoxetine 1 (n = 138 spines/2 dendrites) and fluoxetine 2 (n = 260 spines/4 dendrites). (B, C) Mean spine volume and density in the placebo- or fluoxetine-treated mice. (TIF) [file pone.0147307.s006.tif]

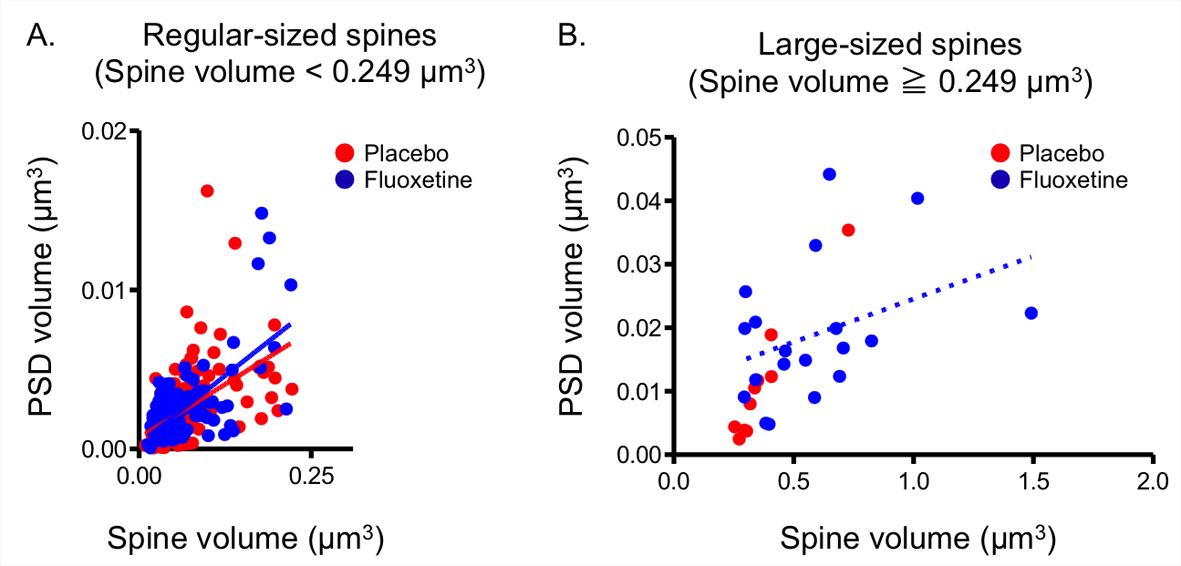

Supplement: S7 Fig — (A) In the regular-sized spines, similar correlations between the PSD and spine volumes were obtained in the placebo- and fluoxetine-treated mice (Placebo: r2 = 0.28, p < 0.0001; Fluoxetine: r2 = 0.44, p < 0.0001) with linear regression analysis. (B) In the large-sized spines of the fluoxetine-treated mice, the PSD volumes were not significantly correlated with the spine volume (r2 = 0.13, p = 0.118). In the placebo-treated mice, a linear regression analysis was not performed because most of the spine volume was distributed in a narrow range (0.249–0.500). (TIF) [file pone.0147307.s007.tif]
